# Trpm8 Expression in Human and Mouse Castration Resistant Prostate Adenocarcinoma Paves the Way for the Preclinical Development of TRPM8-Based Targeted Therapies

**DOI:** 10.3390/biom12020193

**Published:** 2022-01-23

**Authors:** Sacha Genovesi, Riccardo Moro, Beatrice Vignoli, Dario De Felice, Marco Canossa, Rodolfo Montironi, Francesco Giuseppe Carbone, Mattia Barbareschi, Andrea Lunardi, Alessandro Alaimo

**Affiliations:** 1 Department of Cellular, Computational and Integrative Biology (CIBIO), University of Trento, 38123 Trento, Italy; sacha.genovesi@unitn.it (S.G.); riccardo.moro@studenti.unitn.it (R.M.); dario.defelice@unitn.it (D.D.F.); marco.canossa@unitn.it (M.C.); 2 Department of Physics, University of Trento, 38123 Trento, Italy; beatrice.vignoli@unitn.it; 3 Section of Pathological Anatomy, School of Medicine, Polytechnic University of the Marche Region, United Hospitals, 60126 Ancona, Italy; r.montironi@univpm.it; 4 Unit of Surgical Pathology, Santa Chiara Hospital, 38122 Trento, Italy; francescogiuseppe.carbone@apss.tn.it (F.G.C.); mattia.barbareschi@apss.tn.it (M.B.)

**Keywords:** prostate cancer, TRPM8, mouse models, translational research

## Abstract

Metastatic prostate cancer (mPCa) is one of the leading causes of cancer-related mortality in both the US and Europe. Androgen deprivation is the first-line therapy for mPCa; however, resistance to therapy inevitably occurs and the disease progresses to the castration resistant stage, which is uncurable. A definition of novel targeted therapies is necessary for the establishment of innovative and more effective protocols of personalized oncology. We employed genetically engineered mouse models of PCa and human samples to characterize the expression of the TRPM8 cation channel in both hormone naïve and castration resistant tumors. We show that Trpm8 expression marks both indolent (Pten-null) and aggressive (Pten/Trp53 double-null and TRAMP) mouse prostate adenocarcinomas. Importantly, both mouse and human castration-resistant PCa preserve TRPM8 protein expression. Finally, we tested the effect of TRPM8 agonist D-3263 administration in combination with enzalutamide or docetaxel on the viability of aggressive mouse PCa cell lines. Our data demonstrate that D-3263 substantially enhances the pro-apoptotic activity of enzalutamide and docetaxel in TRAMP-C1 e TRAMP-C2 PCa cell lines. To conclude, this study provides the basis for pre-clinical in vivo testing of TRPM8 targeting as a novel strategy to implement the efficacy of standard-of-care treatments for advanced PCa.

## 1. Introduction

Defeating metastatic prostate cancer (mPCa) is a priority to overcome PCa lethality. The identification of new targeted therapies and the development of more effective personalized clinical protocols is crucial to achieve this goal.

In recent years, an unprecedented effort has been devoted to omics approaches aimed at accurately defining the molecular landscape of human PCa [1,2,3]. Besides well-known primary actors, the characterization of a broad spectrum of medium/low-incidence molecular alterations has defined the advanced stages of PCa as a highly heterogeneous disease against which androgen deprivation shows limited efficacy.

Sophisticated targeted therapies against specific oncogenic pathways have been investigated in the clinic during the last decade. Among these, the inhibition of the PI3K/AKT/mTOR pathway is likely one of the most tested, although none of the molecules have yet been approved for clinical use. The most promising results have been obtained by targeting poly-adenosine diphosphate-ribose polymerase (PARP) with the specific inhibitors olaparib and rucaparib, which were approved by the FDA on May 2020 for the treatment of metastatic castration-resistant PCa (mCRPC) characterized by defective DNA Damage Response (DDR) pathway (e.g.,: BRCA1/2 loss-of-function) [4,5].

Proteins hosting a binding pocket for a ligand in their structure represent the “*gold standard*” in pharmacology and the “*holy target”* in oncology. Unfortunately, only a few classes of proteins belong to this category, thus substantially limiting the immediate clinical relevance of much of the knowledge gained through omics studies. In this scenario, an ion channel may represent an interesting alternative. Thanks to the neurosciences, several families of channels are now well-characterized. Organ, tissue, and cell-type specificity, ion selectivity, and biological functions are often very well-known [6,7,8]. Moreover, mechanisms of gating have also been frequently characterized, thus facilitating the identification of natural agonists and antagonists and the consequential development of more potent and selective molecules [9,10,11].

Transient receptor potential cation channel subfamily M (melastatin) member 8 (TRPM8), also known as the cold and menthol receptor 1 (CMR1), was isolated for the first time from the prostate epithelium [12]. RNAseq profiling of normal human tissues defines TRPM8 gene expression as almost exclusive to adult prostate tissue, while immunolocalization with specific antibodies identifies the protein in the luminal compartment of the gland epithelium [13,14,15,16]. The role of TRPM8 in the normal prostate remains controversial. Interestingly, the expression of the channel increases in hormone naïve PCa [15,16], supporting the thesis of a pro-tumorigenic role of the channel, to drastically, and inexplicably, drop in large part of mCRPC [15]. Despite the suggested role of TRPM8 in prostate tumorigenesis [17], in a recent publication we have demonstrated a potent and specific pro-apoptotic response in different cellular models of aggressive primary tumors, lymph node and bone marrow metastases, triggered by the combination of TRPM8 agonists with sub-lethal doses of radio-, hormone-, or chemo-therapy [15].

Here, we characterize, for the first time, Trpm8 expression in mouse prostate epithelium and demonstrate that the Trpm8 protein is abundantly expressed in normal glands, as well as in indolent (*Pten-*null) and lethal (*Pten/Trp53-*double null and TRAMP) mouse prostate adenocarcinomas, though not in neuroendocrine tumors. Importantly, in contrast to human CRPC RNA sequencing data, TRPM8 protein persists in both mouse and human CRPC, suggesting a dichotomy between RNA expression and protein levels in advanced stages of the disease. Finally, a combination of a sub-lethal dose of enzalutamide or docetaxel with Trpm8 agonist D-3263 triggers a potent pro-apoptotic response in aggressive TRAMP-C1 and TRAMP-C2 mouse PCa cells.

This study represents a step forward in the generation of a pre-clinical platform for the in vivo evaluation of the relevance of TRPM8 targeting for the treatment of advanced PCa.

## 2. Materials and Methods

### 2.1. Animals

Wild-type C57BL/6J (JAX #000664) mice were obtained from the Jackson Laboratory. Mice were housed in a certified Animal Facility in accordance with FELASA recommendations, concerning animal welfare, health monitoring and veterinary care, and in compliance with the Directive 2010/63/UE and its Italian transposition D. L.vo 26/2014. All experiments were performed following the ethical guidelines established by the European Communities Council Directive (2010/63/EU) and approved by the Italian Ministry of Health and the University of Trento Animal Welfare Committee (642/2017-PR). Animals were sacrificed at 8 or 18 months or at a humane end point as they displayed signs of morbidity (ataxia, weight loss, and ruffled fur) through CO_2_ inhalation and cervical dislocation for confirmation. The anterior (AP), dorso-lateral (DLP) and ventral prostate (VP) lobes were dissected individually. Finally, the lobes were cut and separated into two portions as follows: one half was fixed in 4% paraformaldehyde (PFA) in PBS and embedded in paraffin for subsequent tissue immunohistochemistry analysis, while the other half was frozen in liquid nitrogen for RNA and proteins extraction.

The *Pten*-null [18], *Pten*;*Trp53*-double null [19] and TRAMP [20] mouse models of hormone naïve and castration resistant PCa were previously generated by the Pandolfi group (Beth Israel Deaconess Medical Center, Harvard Medical School, Boston, MA, USA).

### 2.2. Human and Mouse PCa Samples

Human prostate samples were retrieved from the archives of the Section of Pathological Anatomy of the AOU Ospedali Riuniti of Ancona, Italy. Trans-urethral resection of the prostate (TURP) was performed in patients with urethral neck stenosis due to tumor growth under antiandrogen therapy. The procedure conforms to the provisions of the Declaration of Helsinki.

Mouse prostate tumors (TRAMP, Pten^−/−^ and Pten^−/−^ Trp53^−/−^) were obtained from Pandolfi’s lab (Beth Israel Deaconess Medical Center, Harvard Medical School, Boston, MA, USA).

### 2.3. Cell Lines

Human prostate cell lines RWPE-1 (#CRL-11609), PWR-1E (#CRL-11611) and human prostate carcinoma cell lines LNCaP (#CRL-1740), C4-2 (#CRL-3314), VCaP (#CRL-2876), PC-3 (#CRL-1435), DU-145 (#HTB-81), were purchased from the American Type Culture Collection (ATCC, Manassas, VA, USA) in July 2015. LNCaP and C4-2 cells were cultured in RPMI-1640 (Sigma) supplemented with 10% fetal bovine serum (FBS; Invitrogen), Carlsbad, CA, USA, 1% penicillin/streptomycin (Pen/Strep; Invitrogen, Carlsbad, CA, USA) and 2 mM L-glutamine (Glut; Invitrogen, Carlsbad, CA, USA). VCaP were grown in DMEM (Invitrogen, Carlsbad, CA, USA) containing 10% FBS, 1% Pen/Strep and 2 mM Glut. Both PC-3 and DU-145 cells were cultured in an F-12 medium (Hyclone^TM^;ThermoFisher Sci, Logan, UT, USA) and Alpha MEM Eagle (Lonza, Verviers, Belgium), respectively, either supplemented with 10% FBS, 1% Pen/Strep or 2 mM Glut. Furthermore, RWPE-1 and PWR-1E cells were cultured in keratinocyte serum free medium (KSFM; Invitrogen, Grand Island, NY, USA) supplemented with 0.05 mg/mL bovine pituitary extract (BPE), 5 ng/mL EGF and 1% Pen/Strep. Murine prostate adenocarcinoma TRAMP-C1 and TRAMP-C2 cell lines [21] were kindly provided by Dr. Matteo Bellone (IRCCS San Raffaele Scientific Institute, Milan, Italy) and maintained in DMEM medium supplemented with 10% FBS, 1% Pen/Strep and 2 mM Glut. Plasmid construction and lentiviral transduction for TRPM8 overexpression, used to obtain the line RWPE-1 M8, was performed as described previously [15]. All cells were cultured in a humidified incubator at 37 °C and 5% CO_2_ and were passaged as needed. Cells were tested for specific markers by Western blot and RT-qPCR and routinely tested for Mycoplasma contamination (MycoAlert Mycoplasma Detection Kit, Lonza, Verviers, Belgium).

### 2.4. Chemicals and Drugs

Chemicals and drugs were obtained from the following sources: Docetaxel and Staurosporine from Sigma, St. Louis, MO, USA; D-3263 from Alomone Labs, Jerusalem, Israel; Enzalutamide from Cayman chemicals, Ann Arbor, MI, USA. Enzalutamide (1 mM) was prepared as a stock solution in ethanol; Staurosporine (5 mM), D-3263 (10 mM) and Docetaxel (10 mM) were resuspended in dimethyl sulfoxide (DMSO) to achieve the indicated stock concentrations. All drugs were maintained as stock solutions and stored at −80 °C or −20 °C. In each experiment, the same volume of solvent used for tested drugs and chemicals was added to the control solution.

### 2.5. Immunohistochemistry

Mouse tissues were harvested, immediately washed with PBS and fixed with 4% PFA for 5 h at RT, then collected into histological cassette and subjected to paraffin embedding. Formalin-fixed paraffin-embedded (FFPE) blocks were sectioned at the microtome to obtain 5 µm–thick sections, collected onto glass slides and dried O/N at 37 °C. Slides were dewaxed and re-hydrated through a series of graded ethanol until water. Antigen retrieval was performed using a citrate-based buffer (pH 6.0) (Vector Lab, H3300, Burlington, CA). Slides were incubated in blocking solution (5% FBS + 0.1% Triton-X in PBS) and subsequently stained with TRPM8 primary antibody (Alomone Labs, ACC-049, 1:300, Jerusalem, Israel) O/N at 4 °C, as previously described [15,16]. Slides were washed and incubated with biotin-conjugated secondary antibody (Jackson ImmunoResearch, West Grove, PA, USA) for 1 h at RT, washed again and incubated for 1 h at RT with Avidin-Biotin complex (Vectastain^®^ Elite ABC Peroxidase kit, Vector Labs, Burlington, CA) according to the manufacturer instructions. Finally, samples were incubated with a DAB revelation solution and the reaction was stopped at the desired intensity before mounting the coverslips. For hematoxylin and eosin (H&E) staining, deparaffinized sections were incubated with Gill hematoxylin (Merck, North Shore City, NZ) for 2 min, washed and incubated with eosin Y (Merck, North Shore City, NZ) for 3 min, before mounting the coverslips. Histological observations and images were acquired using an Axio Imager M2 (Zeiss, Jena, Germany).

Immunohistochemical analysis of human prostate samples was performed at the Department of Histopathology (S. Chiara Hospital, Trento, Italy; Prot.:1946 I.D.:112786962) using an automatic immunostainer (BOND-III platform, Leica Biosystems, Wetzlar, Germany). Antigen retrieval was carried out with optimized BOND reagents (Bond epitope retrieval solution 1, Leica Biosystems) at pH 6 for 20 min. BOND compact polymer detection solution (Leica Biosystems) was used for the detection as previously described [15,16]. Images were acquired using an Axio Imager M2 (Zeiss, Jena, Germany).

### 2.6. Western Blot

Cells and tissues were washed in ice-cold PBS twice, and lysed with RIPA buffer (50 mM Tris-HCl, pH 7.5, 150 mM NaCl, 1% Triton X-100, 1% sodium deoxycholate, 1% NP-40) supplemented with protease (Halt^TM^ protease inhibitor cocktail, ThermoFisher Sci) and phosphatase inhibitors cocktail (Phosphatase-Inhibitor-Mix II, solution, Serva) for 30 min at 4 °C. After centrifugation (18,000 g for 20 min at 4 °C), protein concentrations were measured using the BCA assay (Pierce^TM^ BCA protein assay kit, ThermoFisher Sci, Waltham, MA, USA). Equal amounts of protein were loaded and separated by SDS-PAGE, then transferred (300 mA) onto Polyvinylidene fluoride (PVDF) membrane for 2 h (Amersham^TM^ Hybond^TM^, Fisher Scientific, Buckinghamshire, UK) using a wet electroblotting system (Biorad, Hercules, CA, USA). The membranes were blocked with 5% non-fat dry milk or 5% bovine serum albumin (BSA) in TBS-T (50 mM Tris-HCl, pH 7.5, 150 mM NaCl, 0.1% Tween20) for 1 h at RT and then incubated with designated primary antibodies O/N at 4 °C (see below). After three washes in TBS-T, the membranes were incubated with an HRP-conjugated anti-rabbit (Cell Signaling, 7074) or HRP-linked anti-mouse (Cell Signaling, 7076) antibody secondary antibody at 1:1000–1:8000 dilutions in blocking buffer for 1 h at RT. Subsequently, the membranes were washed in TBS-T and immunoreactive bands were detected using ECL LiteAblot plus kit A + B (Euroclone, Milan, Italy) or ECL Select WB Detection Reagent (GE Healthcare, Little Chalfont, UK) with an Alliance LD2 system and software (UVITEC, Cambridge, UK). Immunoblots were performed in at least three independent biological replicates; representative data are shown. The following primary antibodies were used: TRPM8 (Alomone Labs, ACC-049), TRPM8 (Abcam, ab3243), GAPDH (ThermoFisher Sci., MA515738), PARP (Cell Signaling, 9542), Cleaved Caspase-3 (Asp175, Cell Signaling 9661), β-Tubulin (Santa Cruz, sc-5274), HSP90 (Cell Signaling, 4877).

### 2.7. RNA Isolation and End-Point PCR

Frozen tissues and cells were homogenized in Trizol (Trifast, Zymo Research, Irvine, CA, USA), then RNA was extracted using the Direct-zol™ RNA MiniPrep kit (Zymo Research) following the manufacturer’s protocol. The concentration and quality of the RNA were evaluated with a NanoDropTM 2000c spectrophotometer (ThermoFisher Sci, Paisley, UK). For all cases, 1 μg of total RNA was retrotranscribed into cDNA using iScript™ cDNA synthesis Kit (Biorad, Hercules, CA, USA) according to the manufacturer’s protocol. End-point PCR was conducted by amplifying 100 ng of cDNA using the PCRBIO HS Taq Mix Red (PCR Biosystems, London, UK) on a C1000 Touch thermal cycler (Biorad, Hercules, CA, USA). PCR products were loaded on agarose gels and separated by standard gel electrophoresis. DNA gels were imaged with a UV scanner (UVITEC, Cambridge, UK). End-point PCR analyses were performed with at least 3 independent biological replicates, unless stated in the figure legend; representative data are shown. The PCR primers used in the study were Trpm8 (exons 5–6): GTCTAAAGGTGCGTGGATTCT (Fw), CTCTTCTGAGTTCCTGCTGATG (Rv); Trpm8 (exon 17): CTCCTGCTGTTTGCCTATGT (Fw), CATCACAGAAGAGGACGAAGAC (Rv); Trpm8 (exon 13): GATCGCCAAGAACTCCTACAA (Fw), TGCTGCTTCTGTCCTCTTTC (Rv); Trpm8 (exons 11-12): GCAAGACAAGGACAACTGG (Fw), CCTTTATGAGAGCCGTGAAC (Rv); Trpm8 (exons 25-28): TCAAGATCAACACGAAAGCC (Fw), GTTTCTCCCCACAAGCATC (Rv); Trpm8 (exons 14-15): TATGAGACCCGAGCAGTGGA (Fw), CAGGCTGAGCGATGAAATGC (Rv); Gapdh: TGAAGGTCGGAGTCAACGGATTTGG (Fw), CATGTGGGCCATGAGGTCCACCAC (Rv).

### 2.8. Fluorescence Calcium Imaging

Cells were plated on glass coverslips and loaded with 1 μM Fluo-4 acetoxymethyl ester (Fluo-4 AM) for 30 min in darkness in a standard solution containing: NaCl 160 mM, KCl 5.5 mM, CaCl_2_ 1.5 mM, MgSO_4_ 1.2 mM, HEPES 10 mM, glucose 10 mM pH 7.4. Coverslip were then washed with a standard solution for 20 min and placed onto the stage of a direct spinning disk microscope (X-Light V2, Crest optics, Rome, Italy) equipped with a 20X immersion objective [22]. Fluo-4 fluorescence were recorded every 1 s for 60 s before and 540 s after bath application of D-3263 or vehicle. Acquired images were analyzed using CALIMA algorithm [23] manually drawing a circular region of interest (ROI) over cell body. Fluorescence intensity values were converted into ΔF/F ((F_t_–F_0_)/F_0_) calculating F_0_ as the average of fluorescence intensity during baseline recording.

## 3. Results

### 3.1. Trpm8 Expression in Normal Mouse Prostate Epithelium

**Figure 1:**
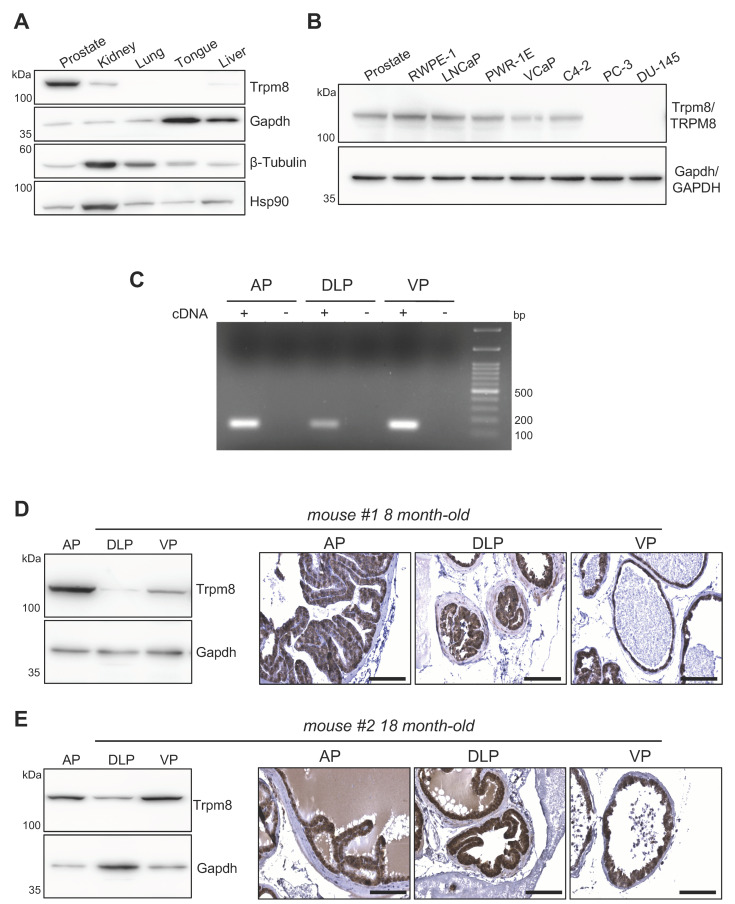
Trpm8 expression in normal mouse prostate. (**A**) Western blot analysis of Trpm8 expression in different mouse organs. Gapdh, β−Tubulin and Hsp90 were used as loading controls. (**B**) Comparison of Trpm8 protein expression levels in mouse prostate and the indicated normal (immortalized) and tumor (metastatic) human prostate cell lines. Gapdh was used as loading control. (**C**) End−point PCR analysis showing Trpm8 mRNA levels in the different mouse prostate lobes. Experiments were performed in quadruplicate. AP, Anterior prostate lobe; DLP, Dorsolateral prostate lobe; VP, Ventral prostate lobe. (**D**,**E**) Representative Western blots (**left**) and immunohistochemical localization (**right**) of Trpm8 in the AP, DLP and VP lobes obtained from an 8 months−old (**D**, mouse #1) and an 18 months-old (**E**, mouse #2) C57BL/6J mice. Gapdh was used as loading control for Western blot analysis. Scale bars, 100 μm.

The prostate gland in the adult mouse male is composed of three distinct lobes located around the urethra, namely the anterior (A), ventral (V), and the dorsolateral lobe (DL) [24]. Each lobe has a distinctive histology. The ventral lobes have moderate to large acini and the luminal spaces presents the smallest amount of infolding relative to the other lobes. The dorsolateral lobes are composed of smaller acini, compared to the other lobes, with moderate infolding and surrounded by a dense stroma. The anterior lobes are characterized by very large acini [24]. Similarly to humans, the mouse prostate contains follicles formed by columnar luminal secretory cells, basal cells and few scattered neuroendocrine cells [24]. As in humans [15,16], the mouse prostate expresses a higher amount of Trpm8 than other murine tissues (Figure 1A), comparable to the levels of the channel found in normal prostate immortalized (RWPE-1 and PWR-1E) and prostate cancer human-cell lines (LNCaP, VCaP, C4-2) (Figure 1B). Semiquantitative RT-PCR shows Trpm8 expression in all the three lobes of mouse prostate, with VP and AP characterized by the highest levels (Figure 1C). Immunoblot analyses of total protein extracts from prostate lobes of 8 month-old mice fully confirmed the PCR data (Figure 1D and Figure S1A-left panel and S1C), while immunolocalization studies define Trpm8 as a specific marker of the luminal compartment of the mouse prostate epithelium (Figure 1D and Figure S1B,C,E). Aging is a crucial event for prostate epithelium homeostasis and a recognized risk factor for human prostate tumorigenesis. An analysis of theTrpm8 status in the prostates of 18 month-old mice demonstrates unchanged protein levels and persistent luminal localization in all three prostate lobes (Figure 1E and Figure S1D).

### 3.2. Trpm8 Marks Hormone Naïve and Castration Resistant Prostate Adenocarcinomas

**Figure 2:**
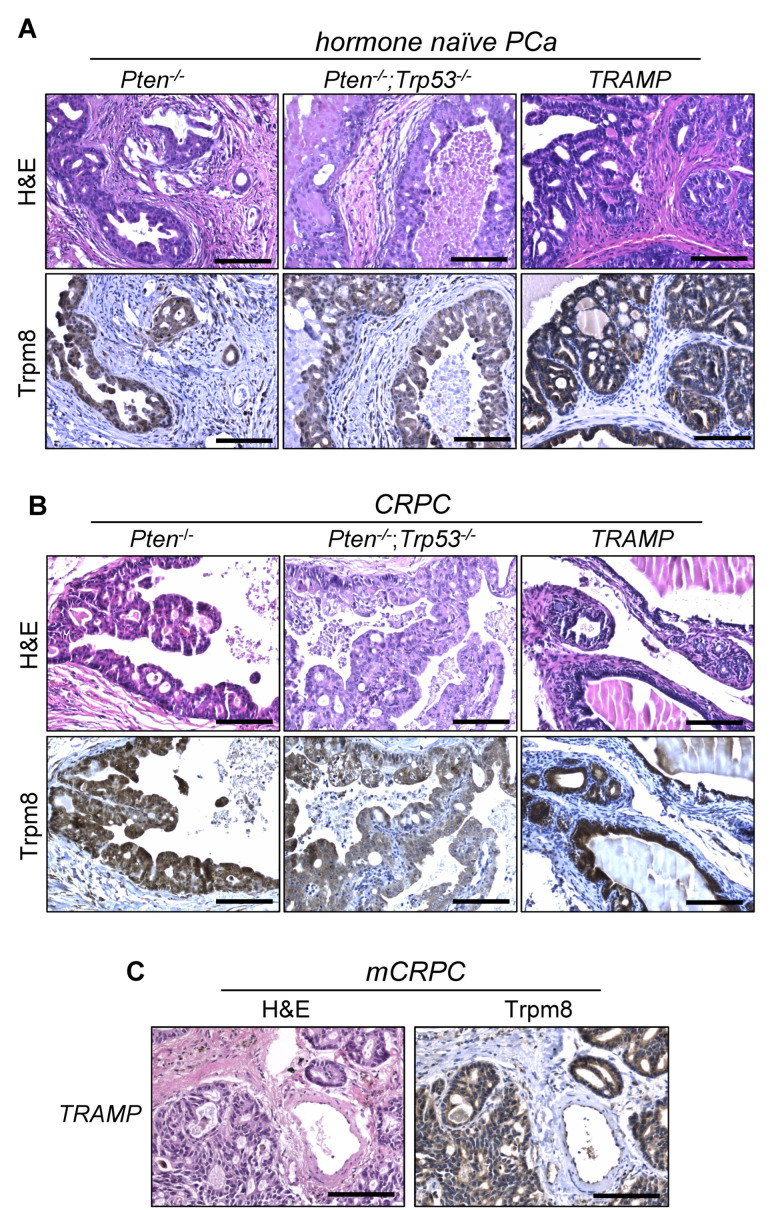
Trpm8 immunostaining in genetically engineered mouse models of hormone−naïve and castration−resistant PCa. (**A**) Representative Haematoxylin-Eosin (H&E) (**above**) and Trpm8 (**below**) staining of hormone naïve adenocarcinoma of Pten−null, Pten;Trp53−double null and TRAMP mice. (**B**) Representative images of H&E (**above**) and Trpm8 (**below**) staining of castration resistant adenocarcinoma of Pten−null, Pten; Trp53−double null and TRAMP mice. (**C**) H&E (**left**) and Trpm8 (**right**) staining of a castration resistant PCa lung metastasis of a castrated TRAMP mouse. Scale bars, 100 μm.

Despite a certain level of inter-tumor heterogeneity, TRPM8 immunolocalization shows a well-conserved expression of the channel in human hormone-naïve primary PCa and proximal lymph node metastases [15,16]. To parallel these findings in both indolent and aggressive forms of mouse prostate tumors, we took advantage of three classical genetically engineered mouse models of PCa, whereby the tumorigenic process in the prostate epithelium is driven by conditional loss of Pten (Pb-Cre4;Pten^flox/flox^), Pten and Trp53 (Pb-Cre4;Pten^flox/flox^;Trp53^flox/flox^), or the overexpression of the large T antigen of SV40 (TRAMP) [19,25,26]. As is known, Trpm8 protein is robustly expressed by cancer cells of both indolent (Pten-null) and aggressive (Pten;Trp53-double null and TRAMP) forms of hormone naïve adenocarcinoma (Figure 2A), though not neuroendocrine (Figure S2), mouse PCa.

**Figure 3:**
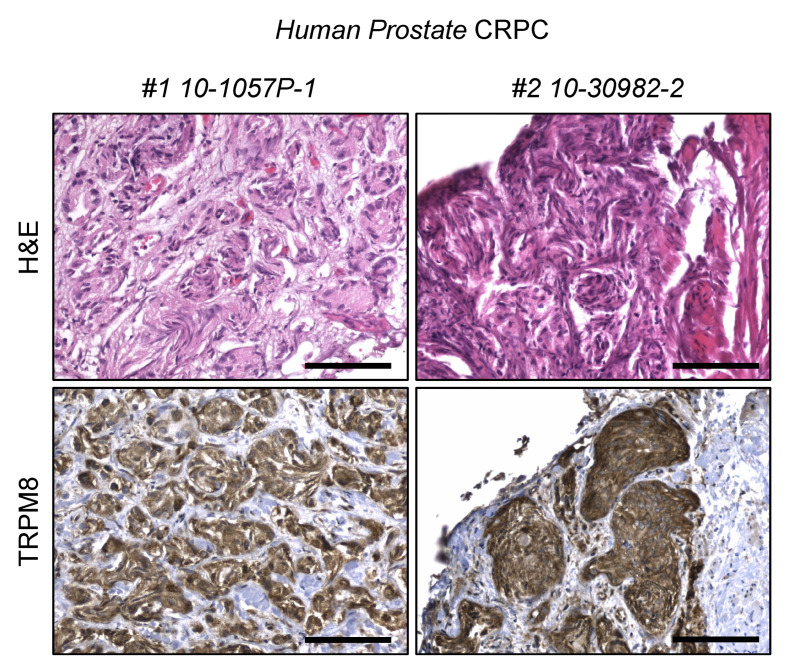
Trpm8 marks human castration resistant PCa samples. H&E (**above**) and immunostaining for TRPM8 (**below**) in human CRPC specimens. Representative images are shown. Scale bars, 100 μm.

As in humans, mouse-prostate tumorigenesis is supported by androgens. However, tumor response to androgen ablation is highly dependent on the molecular mechanisms driving the tumorigenic process [26]. To investigate Trpm8 status in castration resistant mouse PCa (moCRPC), immunolocalization studies were performed in Pten-null, Pten;Trp53-double null and TRAMP mouse prostate tumors collected two months after bilateral orchiectomy [26]. Pten-null prostate tumors are very sensitive to testosterone levels and shrink significantly after androgen ablation, although resistance inevitably occurs later on. Differently, Pten;Trp53-double null and TRAMP prostate tumors are intrinsically refractory to androgen deprivation, and continue growing exponentially despite of castrated amount of testosterone [26]. Androgen ablation shows no relevant consequences on Trpm8 expression, which remains consistently expressed in castration-resistant tumors developed in the three mouse models enrolled in the study (Figure 2B,C). In contrast with this finding, transcriptional profile analyses of human castration resistant PCa (huCRPC) collected in the TCGA repository describe a sharp decline of TRPM8 mRNA levels in most of samples [15]. To reconcile this apparent paradox, we analyzed TRPM8 protein expression in human CRPC samples. TRPM8 immunolocalization reveals a robust expression of the channel in 8 out of 10 human castration resistant tumors (Figure 3 and Figure S3), which suggests a substantial difference between mRNA levels and protein amounts of TRPM8 in human CRPC.

### 3.3. Trpm8 Agonist D-3263 Favors Cancer Cell Killing Efficacy of Chemo and Hormone Therapy

**Figure 4:**
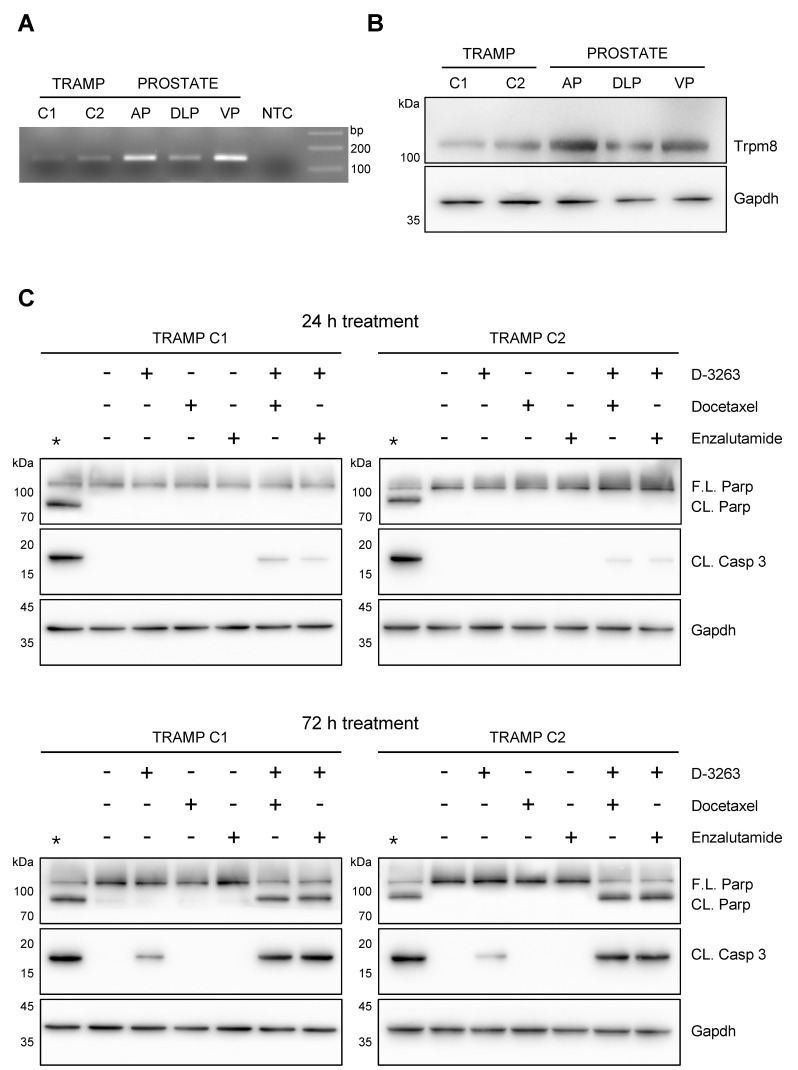
Cooperative effect of the Trpm8 agonist D−3263 with Enzalutamide or Docetaxel in TRAMP PCa cell lines. (**A**,**B**) End−point PCR (**A**) and Western blotting (**B**) analyses showing the expression of Trpm8 in TRAMP C1 and C2 cell lines compared to normal mouse prostate AP, DLP and VP lobes. Gapdh was used as loading control. Experiments were performed in quadruplicate. (**C**) Western blotting analysis showing molecular signature of apoptotic cell death (Caspase−3 and Parp cleavage). TRAMP C1 and TRAMP C2 cells were treated with D−3263 (1 μM), Docetaxel (5 nM), Enzalutamide (1 μM), D-3263 + Docetaxel, or D-3263 + Enzalutamide for 24 or 72 h. Untreated cells were used as control. Gapdh was used as loading control. Experiments were performed in triplicates. * Protein extracts from cells treated with Staurosporine (1 μM, 6 h) were used as positive control.

We recently demonstrated that the administration of the potent TRPM8 agonists WS-12 in combination with sub-lethal doses of radio-, chemo- or hormone-therapy triggers a rapid and considerable apoptotic response in 2D and 3D models of hormone naïve primary and metastatic human PCa [15]. Thus, we decided to test the same strategy in the mouse prostate cancer cell lines TRAMP-C1 and TRAMP-C2 [21,27]. RT-qPCR and Western blot analyses detect Trpm8 mRNA and protein expression in both cell lines (Figure 4A,B). Then, the Trpm8 response to D-3263 was evaluated by measuring cytosolic calcium accumulation through quantitative Fluo-4 imaging. Similar to previous observations in the human metastatic PCa cell line LNCaP stimulated with the WS-12 [15], Fluo-4 imaging shows no detectable changes in cytosolic calcium concentration in both TRAMP-C1 and TRAMP-C2 cell lines treated with D-3263 (Figure S4). Concordantly, immunoblot analyses for Caspase 3 and Parp cleavage demonstrate no signs of cytotoxicity after 24 h treatment with D-3263, as well as with sub-lethal doses of docetaxel or enzalutamide (Figure 4B), while low levels of cytotoxicity appear in both cell lines after 72 h of D-3263 treatment (Figure 4C). A combination of D-3263 with either docetaxel or enzalutamide promotes a mild but consistent cleavage of Caspase 3 in TRAMP-C1 and TRAMP-C2 mouse PCa cell lines at 24 h (Figure 4B), while inducing a massive apoptotic response in both TRAMP-C1 and TRAMP-C2 cell lines at 72 h (Figure 4C). Similar to what we observed with the WS-12 treatment in human LNCaP cells [15], we speculate that D-3263 may promote a slow but progressive increase in cytosolic [Ca^2+^] in mouse PCa cell lines, thus becoming cytotoxic over time.

## 4. Discussion

Prostate cancer is a very common human disease, and the clinical management of PCa patients represents a major effort world-wide [28,29]. Nowadays, almost 9 out of 10 newly diagnosed PCa cases are organ-confined tumors that can be successfully controlled through radiotherapy or surgery [30]. Different prognosis is generally associated with locally advanced/high-risk PCa, which has a high probability of progressing to the metastatic stage of the disease, which unfortunately remains incurable [31]. Finally, less than 40% of patients with metastatic PCa at diagnosis will survive 5 years of their disease [32]. If the identification of reliable biomarkers and the development of more sophisticated diagnostic tools will improve the detection of neoplastic prostate lesions at their earliest stages, then the establishment of a more effective clinical protocol for the treatment of advanced stages of the disease is a crucial oncological need. Androgen deprivation is effective in the vast majority of cases, but, inevitably, cancer cells acquire resistance. No effective treatments have been established for metastatic castration resistant PCa (mCRPC), and the clinical protocol foresees generic chemotherapy, mainly as palliative care.

In a recent publication [15], we explored the possibility to consider ion channels as candidate targets for innovative approaches of precision oncology in PCa. We focused our attention on TRPM8, a cation channel abundantly expressed in the luminal compartment of the prostate epithelium, whose permeability is three times higher for calcium than potassium or sodium [33,34]. In that study, we concluded that TRPM8-driven calcium cytotoxicity synergizes with radio-, chemo- or hormone therapy to establish a lethal condition in prostate cancer cells [15]. Although promising, these results represent the first step in the pre-clinical evaluation of a novel therapeutic approach. To move forward with our analysis and test of TRPM8 targeting in vivo in mouse models of PCa, here we described a careful characterization of Trpm8 expression in normal and malignant mouse prostate tissues. We demonstrate that Trpm8 expression is enriched in mouse prostate luminal cells, and, as in humans, it marks hormone naïve prostate cancer cells independent of the genetic and molecular drivers that promote tumorigenesis. According to RNAseq studies [15], TRPM8 expression declines drastically with tumor progression to the castration-resistant stage of the disease. However, we found a robust expression of Trpm8 protein in castration-resistant mouse PCa, both primitive and metastatic lesions. This unexpected result was further confirmed by immunolocalization studies on human CRPC samples that showed high TRPM8 staining in 8 out of 10 cases. The dichotomy between mRNA expression and protein levels of TRPM8 is not surprising, since this condition was previously described in the immortalized human prostate cell lines RWPE-1 and PWR-1E [15,35]. A very interesting observation suggests a translational-independent pro-inflammatory role of the mRNA molecule of TRPM8, which might contribute to explain why aggressive prostate cancer cells minimize the amount of TRPM8 mRNA while leaving unaltered the level of the protein (Alaimo et al., *in preparation*). Identification of TRPM8/Trpm8 protein in both human and mouse CRPC is an important finding that moves forwards the possible relevance of studying TRPM8 targeting in PCa.

In this scenario, the massive apoptotic response induced in TRAMP-C1 and TRAMP-C2 mouse PCa cell lines by the co-administration of the D-3263 channel agonist with sub-lethal doses of either enzalutamide or docetaxel, paves the way for dedicated in vivo pre-clinical studies. Importantly, the C57BL/6J genetic background of the TRAMP-C1 and TRAMP-C2 cell lines will allow us to test the gating of Trpm8 in mouse models of hormone-naïve and therapy resistant PCa generated through the orthotopic transplantation of these cells into immune competent syngeneic recipient mice.

## 5. Conclusions

The treatment of advanced PCa remains a major issue in oncology. Hormone therapy is the main therapeutic option for metastatic PCa patients. However, this treatment inevitably fails, and chemotherapy for castration resistant tumors has only a palliative effect. Novel therapeutic options are essential to counteract metastatic disease progression and lower CRPC mortality. This study lays the foundations for testing in vivo, in mouse models of aggressive and hormone therapy refractory PCa, and the therapeutic relevance of combining potent TRPM8 channel agonists with standard-of-care clinical approaches.

## Data Availability

The data presented in this study are available in this article. The authors are available for any additional inquiries related to the data and the procedures.

## Supplementary Materials

The following supporting information can be downloaded at: https://www.mdpi.com/article/10.3390/biom12020193/s1, Figure S1: Characterization of Trpm8 expression in normal mouse prostate, Figure S2: Trpm8 protein is expressed by adenocarcinoma but not by neuroendocrine PCa cells in TRAMP models, Figure S3: Characterization of Trpm8 expression in human castration resistant PCa samples, Figure S4: Calcium imaging in prostate cell lines, Figure S5: Uncropped Western blot figures.
