# Supplementary material for: Trpm8 Expression in Human and Mouse Castration Resistant Prostate Adenocarcinoma Paves the Way for the Preclinical Development of TRPM8-Based Targeted Therapies
Source: Biomolecules. 2022 Jan 23;12(2):193. doi: 10.3390/biom12020193 (PMC8961668; doi:10.3390/biom12020193)
Supplement: Supplementary file 1 [file biomolecules-12-00193-s001.zip › biomolecules-1549981-supplementary.pdf]

Supplementary Materials: Trpm8 expression in human and mouse castration resistant prostate adenocarcinoma paves the way for the preclinical development of TRPM8-based targeted therapies

Sacha Genovesi [1], Riccardo Moro [1], Beatrice Vignoli [2], Dario De Felice [1], Marco Canossa [1], Rodolfo Montironi [3], Francesco Giuseppe Carbone [4], Mattia Barbareschi [4], Andrea Lunardi [1,*,†], and Alessandro Alaimo [1,*,†]

[1] Department of Cellular, Computational and Integrative Biology (CIBIO), University of Trento, 38123 Trento, Italy; sacha.genovesi@unitn.it (S.G.); riccardo.moro@studenti.unitn.it (R.MOR.); dario.defelice@unitn.it (D.D.F.); marco.canossa@unitn.it (M.C.)
[2] Department of Physics, University of Trento, 38123 Trento, Italy; beatrice.vignoli@unitn.it (B.V.)
[3] Section of Pathological Anatomy, Polytechnic University of the Marche Region, School of Medicine, United Hospitals, 60126 Ancona, Italy; r.montironi@univpm.it (R.MON.)
[4] Unit of Surgical Pathology, Santa Chiara Hospital, 38122 Trento, Italy; francescogiuseppe.carbone@apss.tn.it (F.G.C.); mattia.barbareschi@apss.tn.it (M.B.)
* Correspondence: andrea.lunardi@unitn.it (A.L.); alessandro.alaimo@unitn.it (A.A)
† A.L. and A.A. contributed equally as last authors.

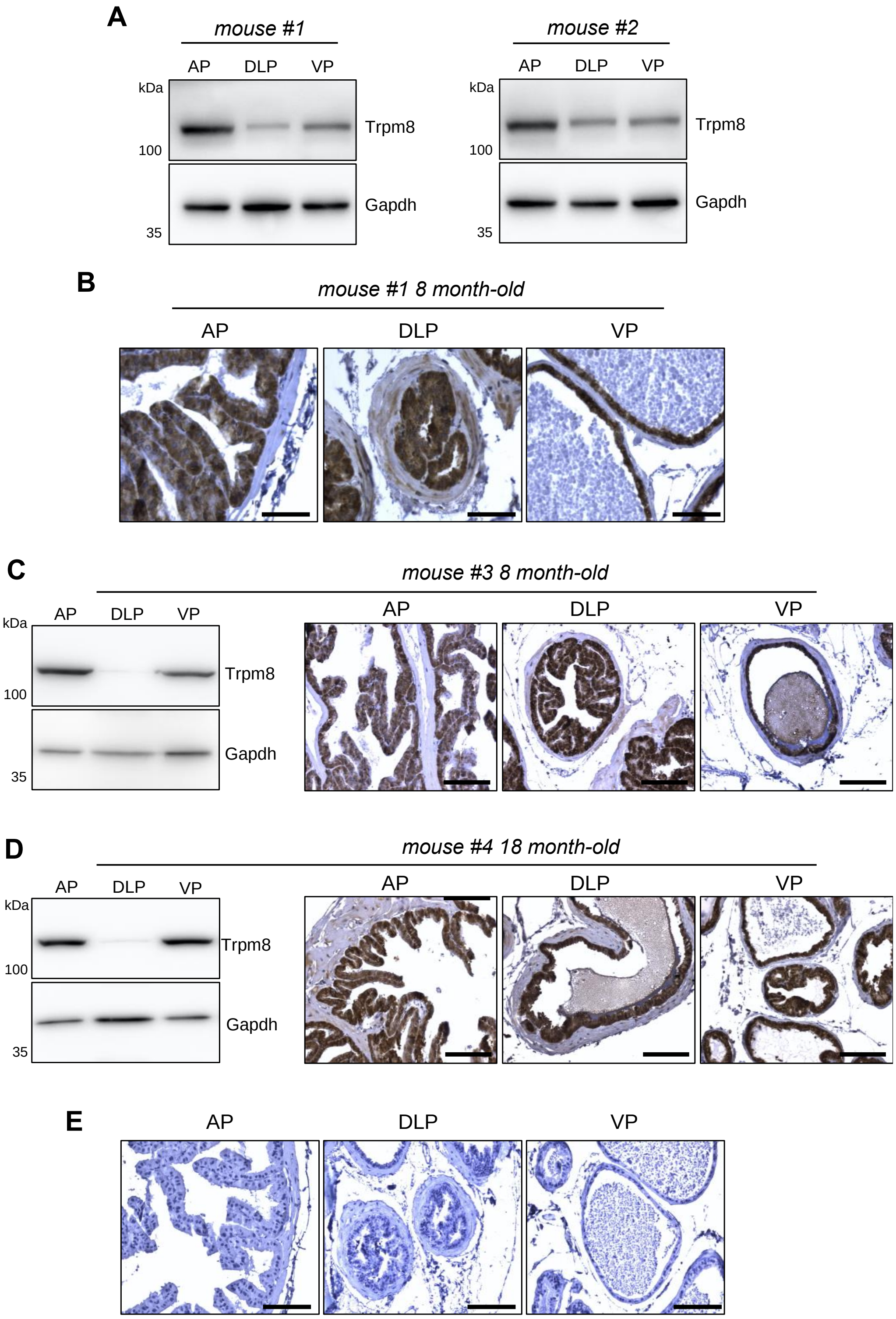

Figure S1. Characterization of Trpm8 expression in normal mouse prostate. (**A**) Western blot analysis of Trpm8 expression using a primary anti-TRPM8 antibody from Abcam (ab3243) which recognizes a different epitope compared to Alomone (ACC-049). Proteins were extracted from AP, DLP and VP lobes of 8 months-old (mouse #1) and 18 months-old (mouse #2) (related to Figure 1D and 1E, left). Gapdh was used as loading control. (**B**) 40x magnification of Figure 1D, right (mouse #1). Scale bars, 50 µm. (**C-D**) Representative Western blots (left) and immunohistochemical localization (right) of Trpm8 in the AP, DLP and VP lobes obtained from a 8 months-old (**C**, mouse #3) and a 18 months-old (**D**, mouse #4) C57BL/6J mice. Gapdh was used as loading control for Western blot analysis. Scale bars, 100 µm. (**E**) Sections of prostate lobes (mouse #1, related to Figure 1D) incubated with the secondary antibody alone confirming anti-TRPM8 antibody (ACC-049, Alomone labs) specificity.

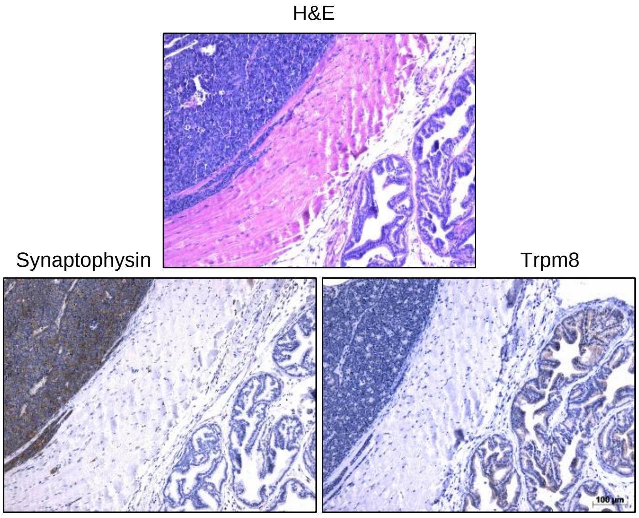

Figure S2. Trpm8 protein is expressed by adenocarcinoma but not by PCa cells in TRAMP models. Representative H&E (above) and immunostaining for Synaptophysin (below, left) and Trpm8 (below, right) of TRAMP mouse model specimens showing the absence of Trpm8 in neuroendocrine PCa cells. Scale bars, 100 µm.

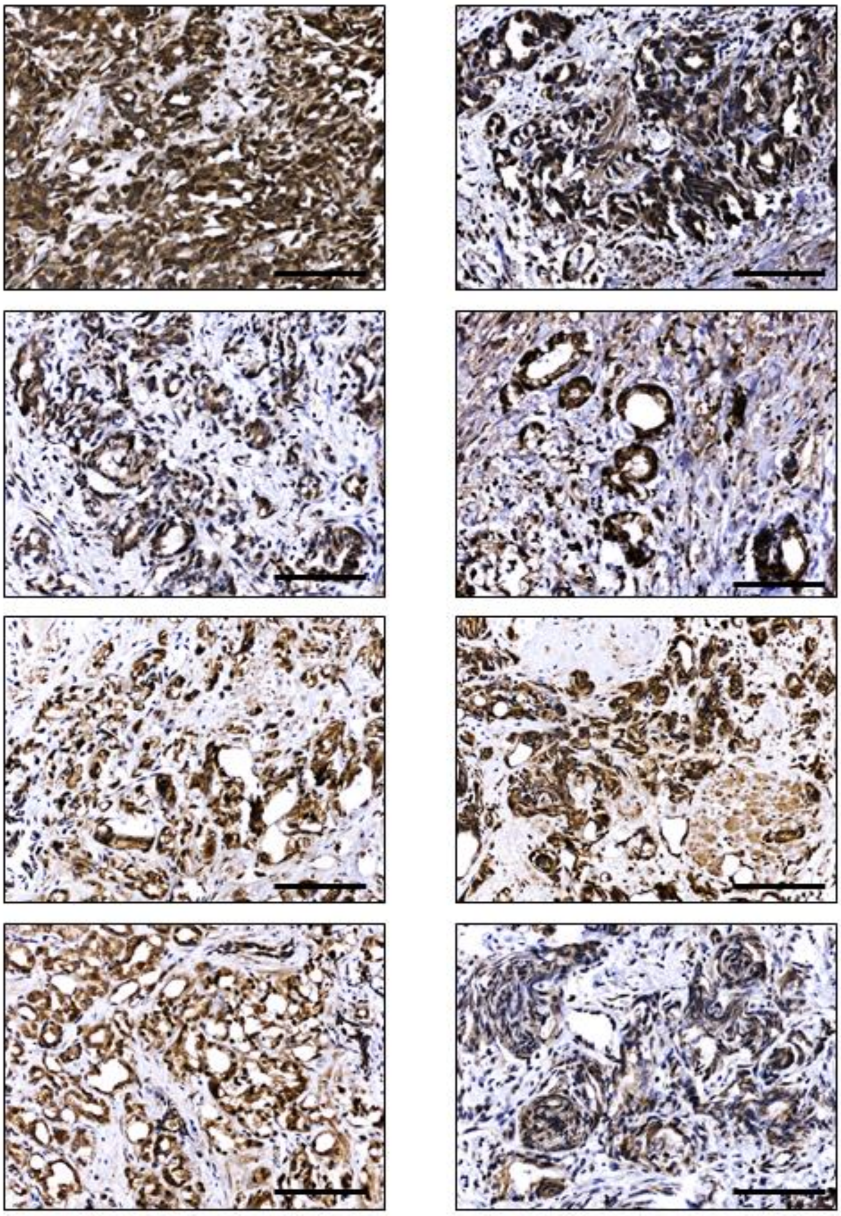

Figure S3. Characterization of Trpm8 expression in human castration resistant PCa samples. Immunostaining for TRPM8 in human CRPC specimens. Scale bars, 100 µm

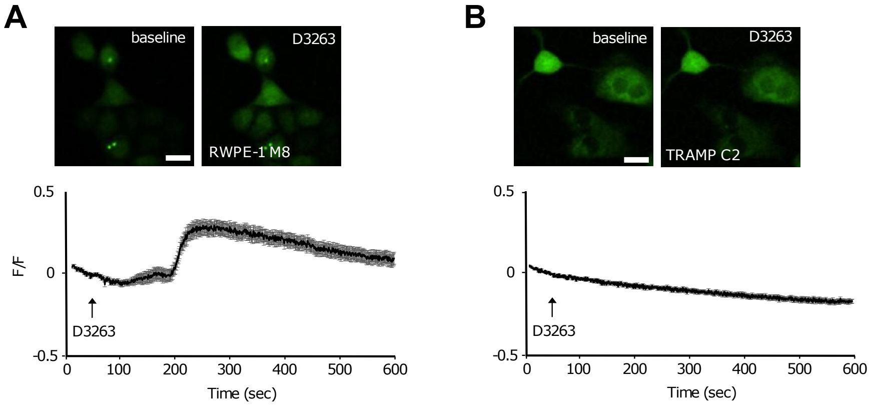

Figure S4. Fluorescence Calcium Imaging in prostate cells. (**A**) Panels show representative spinning disk confocal images of the Fluo-4 fluorescence for RWPE-1 M8 cells before (10 s) and after (180 s) treatment with D-3263. Histogram depicts the corresponding ΔF/F- values. Data are mean ± SEM (n=57 cells). (**B**) Panels show representative spinning disk confocal images of the Fluo-4 fluorescence for TRAMP C2 cells before (10 s) and after (180 s) treatment with D-3263. Histogram depicts the corresponding ΔF/F- values. Data are mean ± SEM (n=58 cells). Scale bars, 15 μm.

Figure 1A

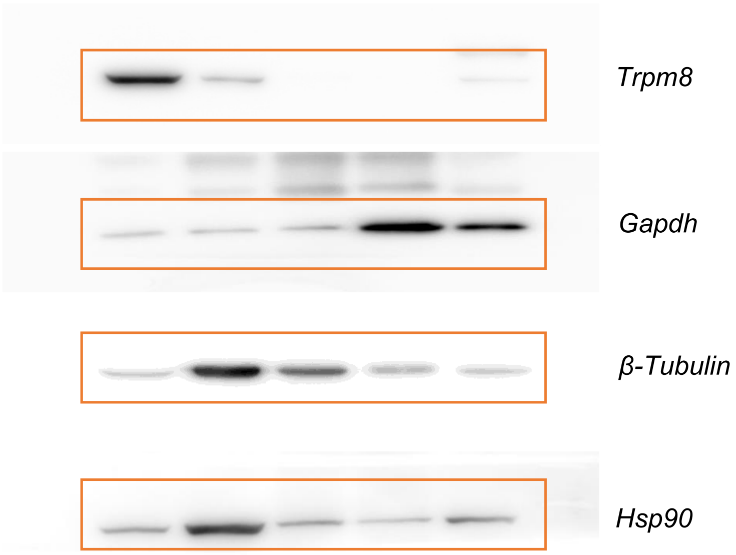

Figure 1B

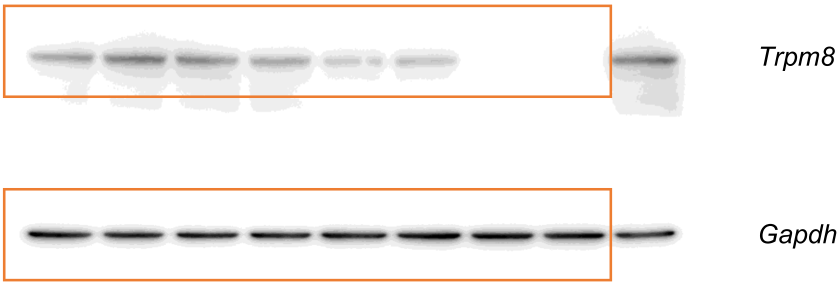

Figure 1C Figure S1C

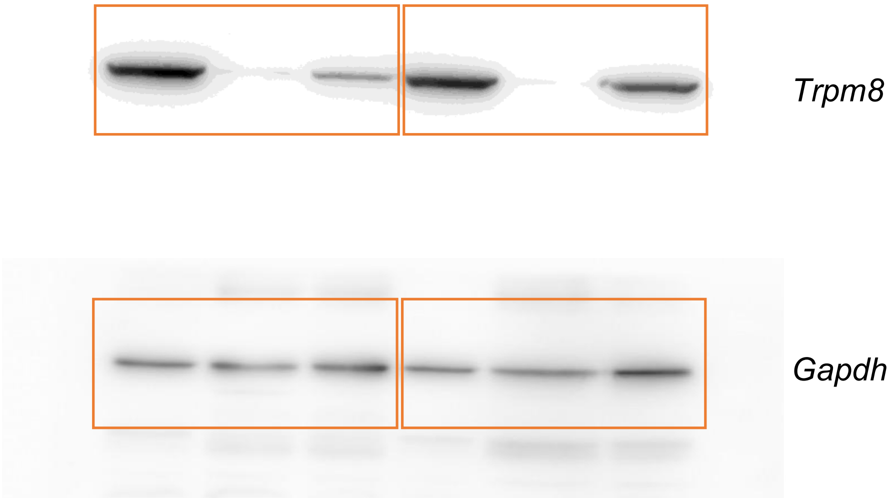

Figure S1D

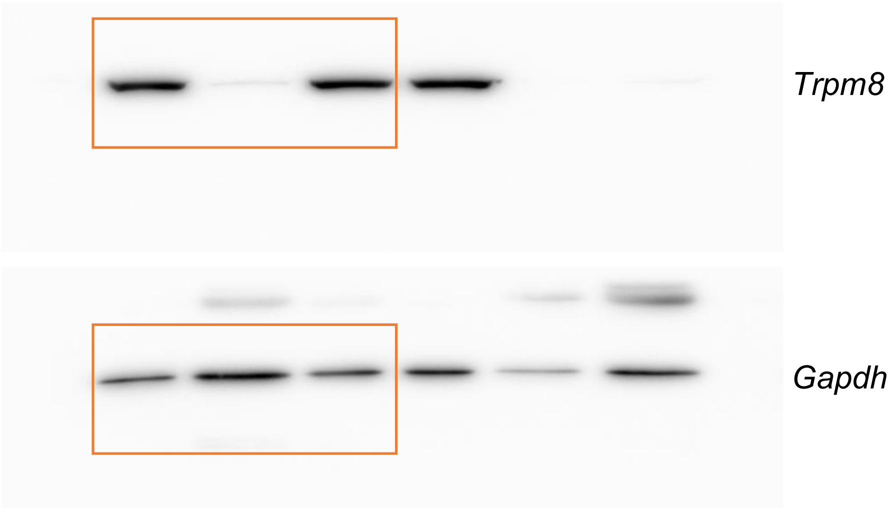

Figure 1D

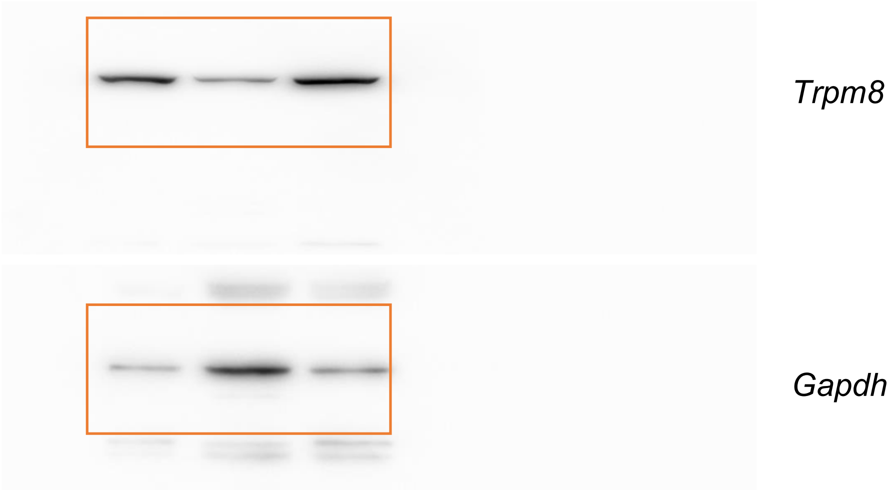

Figure S1A

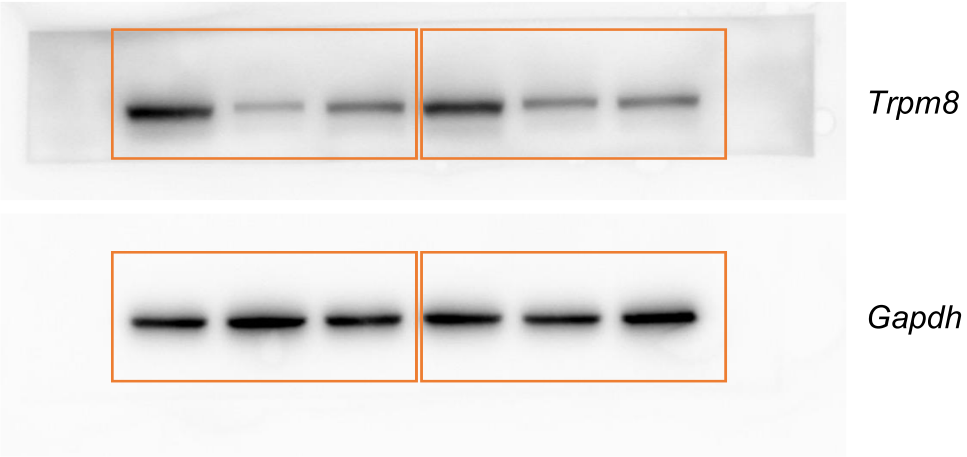

Figure 4B

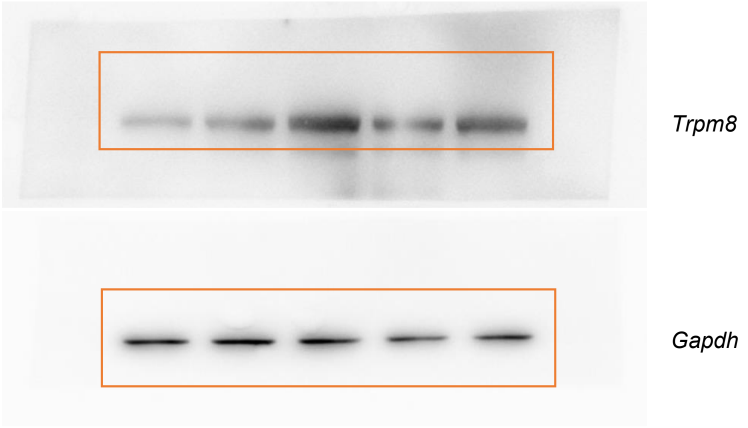

Figure 4C

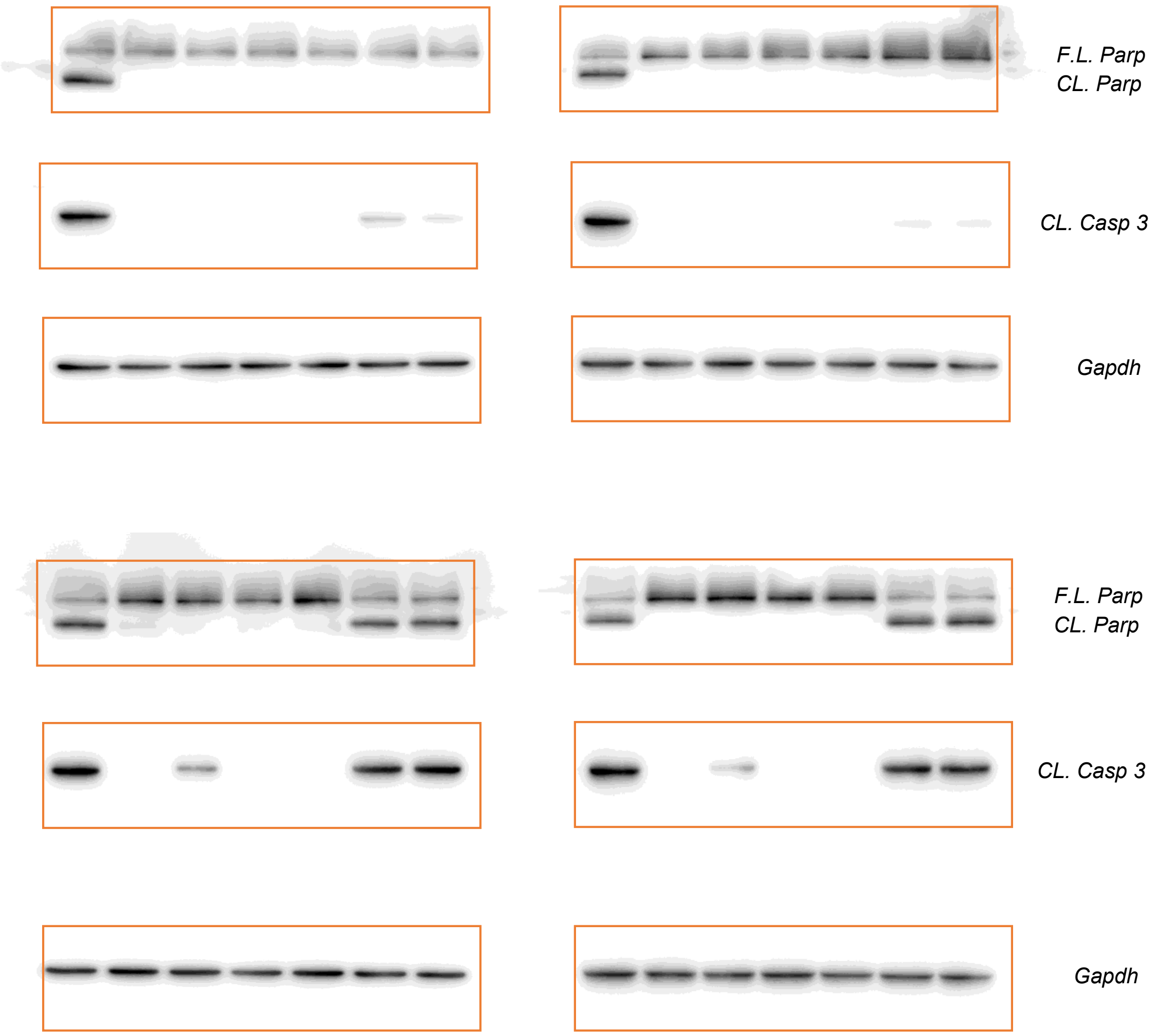

Figure S5. Uncropped western blot figures.
